# Content Validation of a Professionalism in Public Health Scale (PPHS) for Pakistani Public Health Professionals: An Expert Panel Study

**DOI:** 10.12669/pjms.42.5.14981

**Published:** 2026-05

**Authors:** Syed Fawad Mashhadi, Rukhsana Ayub, Khadija Qamar, Muhammad Alamgir Khan

**Affiliations:** 1 Syed Fawad Mashhadi, PhD Department of Community Medicine & Public Health, Army Medical College, National University of Medical Sciences, Rawalpindi, Pakistan; 2 Rukhsana Ayub, FCPS Department of Health Professions Education, National University of Medical Sciences, Rawalpindi, Pakistan; 3 Khadija Qamar, FCPS Department of Medical Education Army Medical College, National University of Medical Sciences, Rawalpindi, Pakistan; 4 Muhammad Alamgir Khan, FCPS Department of Physiology, Army Medical College, National University of Medical Sciences, Rawalpindi, Pakistan

**Keywords:** Content Validity, Expert Panel, Professionalism, Public Health, Pakistan, Psychometrics, Scale Development

## Abstract

**Objective:**

To assess the content validity of the Professionalism in Public Health Scale (PPHS), developed for Pakistani public health professionals, through a structured expert-panel review.

**Methodology:**

A cross-sectional expert-panel study was conducted from Sept 2025 - Dec 2025 at the National University of Medical Sciences (NUMS), Rawalpindi. Ten experts with experience in public health practice, education, and research independently reviewed an initial pool of 45 items generated through theoretical synthesis and structured literature review. Each item was rated for relevance (4-point scale), necessity (3-point scale), and clarity (4-point scale), with space for narrative suggestions. Quantitative content validity indices were calculated (item-level CVI and scale-level CVI/Ave), along with Content Validity Ratio (CVR) to assess item essentiality. Written qualitative feedback was analysed in parallel and used to guide item refinement and removal decisions.

**Results:**

PPHS demonstrated strong scale-level content validity (S-CVI/Ave = 0.88), with consistently high domain-level indices (S-CVI/Ave = 0.83–0.91). Based on combined quantitative thresholds and narrative feedback, nine items were removed and several were revised for clarity and construct alignment, yielding a final 36-item scale (80% retention). Key expert suggestions centered on improving wording and specificity, addressing response-scale suitability, and correcting double-barreled statements.

**Conclusion:**

This expert review provides evidence of excellent content validity for the 36-item PPHS and supports progressing to field testing and further psychometric evaluation in Pakistani public health settings.

## INTRODUCTION

Across the health professions, professionalism is regarded as a fundamental value, embodying the principles of ethics, accountability, continuous learning, collaboration, and community service.^1^,^2^ While professionalism research is well established in the clinical fields, less attention has been paid to what a public health professional’s behavior should entail when the focus is a population rather than an individual.

Public health professionals operate within and across multiple disciplines and sectors, manage scarce resources, and implement preventive choices that may involve trade-offs between individual and collective levels.^3^,^4^ Consequently, tools developed for clinical settings may overlook important professional attributes that relate to public health, especially the roles of advocacy, systems thinking, and community involvement.

Available evidence indicates that these gaps remain significant. Veloski and colleagues, in a review of instruments used to assess professionalism, found that the majority were developed for the clinical training and practice.^5^ Li et al. confirmed that this clinical dominance persists across instrument development.^6^ The lack of context appropriate instruments is particularly limiting in low- and middle-income countries, where valid assessment tools are essential for workforce development and health system strengthening.

More contextual considerations apply to Pakistan. Professional decision-making is influenced by religious-ethical considerations and/or the organizational hierarchy, together with resource constraints and an epidemiological situation, that emphasises preventive and community-focused approaches.^7^,^8^A tool developed in Pakistan, therefore, stands a better chance of portraying the behaviors and attitudes of the public health community.^9^ In scale development, content validity evidence is gathered when experts assess whether the item pool sufficiently captures the construct in language that the target audience can understand and relate to.^10^-^12^ Within Messick’s unified validity perspective, this work bolsters score interpretation by demonstrating the association of the items to the theoretical constructs being measured.^13^

The purpose of this study was to document the content validity of the PPHS via a structured expert review integrating quantitative measures and qualitative feedback to assess and improve the item pool before field testing.^14^

## METHODOLOGY

This cross-sectional expert panel study was conducted at the National University of Medical Sciences (NUMS), Rawalpindi, Pakistan, from September to December 2025 as part of the broader validation for PPHS.

### Ethical approval:

It was obtained from the Institutional Review Board of NUMS (Approval No. 06/IRB&EC/NUMS/103; Dated 23 September 2025).

The item pool was generated by a structured literature review of PubMed, CINAHL, PsychINFO, Scopus regional databased (Index Medicus, WHO IRIS) using terms including “public health professionalism’ ‘professionalism assessment scale’ and ‘public health workforce competency measurement’. The review confirmed the absence of any validated professionalism scale specifically designed for public health professionals. Items were then generated through a systematic construct mapping process operationalizing three complementary sociological theories into measurable behavioural indicators (Table-I); Halls Trait theory provided structural attributes (Autonomy, self-regulation, professional commitment), Social Contract Theory contributed relational dimensions (public trust, ethical obligation, accountability) and Symbolic Interactionism informed dynamic aspects (reflexivity, contextual adaptability, identity negotiation). Six domains were specified:

- Ethical Stewardship and Integrity,
- Accountability and Professional Duty,
- Competence and Reflective Growth,
- Interprofessional Collaboration and Respect,
- Advocacy and Community Orientation, and
- Self-Awareness and Adaptive Professionalism. We drafted 45 items across these domains, designed for response on a 5-point Likert scale (1 = Strongly disagree to 5 = Strongly agree).

**Table-I:** Theory to Domain Operationalization Matrix.

Theory	Key Constructs	PPHS Domain(s)	Example
Hall’s Trait Theory (1968)	Professional autonomy, sense of calling, self-regulation, organizational affiliation	D2: Accountability & Professional Duty; D3: Competence & Reflective Growth	‘I hold myself accountable for meeting professional standards’; ‘I actively seek feedback to improve my practice’
Social Contract Theory (Cruess & Cruess, 2006)	Reciprocal obligations, public trust, ethical practice, stewardship of resources	D1: Ethical Stewardship & Integrity; D5: Advocacy & Community Orientation	‘I prioritise public welfare over personal interests’; ‘I advocate for equitable access to health services’
Symbolic Interactionism (Mead, 1934; Blumer, 1969)	Socially constructed meaning, reflexive identity, contextual behavior	D4: Interprofessional Collaboration & Respect; D6: Self-Awareness & Adaptive Professionalism	‘I adapt my communication to different professional contexts’; ‘I recognise when assumptions influence my judgment’
Messick’s Unified Validity Theory (1989, 1995)	Five sources of validity evidence, construct representation	Overarching validation framework (all phases)	Content validation (this study); response process, internal structure (planned)

Ten content experts were purposively recruited. We followed Lynn’s guidance on minimum panel size and Polit and Beck’s recommendation that panels of 6–10 experts balance feasibility with reliability.^12^,^14^ Eligibility criteria were (a) at least 10 years of experience in public health practice, education, research, or policy; (b) an advanced qualification in public health or medical education (e.g., PhD, MPH, MPhil, MHPE); (c) evidence of scholarship relevant to public health, professionalism, or measurement; and (d) familiarity with Pakistani public health systems (required for at least 6 of 10 experts). To mitigate selection bias, the panel was constituted from various institutions of Pakistan to achieve diversity in professional role and organisational type (federal, provincial and NGOs with no more than two from any single institution). All ratings were submitted independently and kept confidential to ensure unbiased judgement. Experts received a structured validation pack by email, including study background, the conceptual framework and domain map, the full 45-item pool, and a rating form for each item with space for written comments and wording suggestions.

Each item was rated on three dimensions:-

- *Relevance (4-point scale):* 1=Not relevant, 2=Somewhat relevant, 3=Quite relevant, 4=Highly relevant
- *Necessity (3-point scale):* 1=Not necessary, 2=Useful but not essential, 3=Essential
- *Clarity (4-point scale):* 1=Very unclear, 2=Unclear, 3=Clear, 4=Very clear
- Experts were asked to complete the review within two weeks. A reminder message was sent after one week.

### Quantitative analysis comprised the following indices:

- Item-level Content Validity Index (I-CVI): the proportion of experts rating an item as quite/highly relevant (3 or 4). For a 10-expert panel, I-CVI ≥0.78 was used as the primary criterion for adequate relevance.^9^
- Scale-level CVI (S-CVI/Ave): the mean of all I-CVI values across items, S-CVI/Ave ≥0.80 indicates strong content validity.^9^
- Content Validity Ratio (CVR): calculated using Lawshe’s formula, CVR = (ne − N/2) / (N/2), where ne is the number of experts rating the item as “essential”. For N = 10 experts, the critical CVR value is 0.62.^15^ CVR ≥0.62 indicates that expert consensus on the item’s essentiality exceeds what would be expected by chance alone.

### Mean clarity score:

calculated as the arithmetic mean of all expert clarity ratings for each item. A threshold of ≥3.0 (corresponding to ‘Clear’ on the 4-point scale) was applied; items scoring below this threshold were flagged for mandatory rewording regardless of their relevance or necessity ratings.

While the validation approach is guided by Messick’s Unified validity framework, the content validity procedures employed in this study i.e. systematic expert assessment of relevance, comprehensiveness and comprehensibility - are also consistent with the COSMIN content validity, thereby aligning with contemporary measurement standards. Written comments were reviewed and organized into themes (wording/clarity, conceptual fit, cultural/contextual appropriateness, redundancy, and response-format issues) and used to guide item revision decisions. Items were generally retained when they met all three quantitative thresholds (I-CVI ≥0.78, CVR ≥0.62, and mean clarity ≥3.0) and did not attract major qualitative concerns. Items were revised when quantitative indices were acceptable but experts identified clarity or wording problems. Items were removed when they failed relevance or necessity thresholds, were consistently judged redundant, or were considered outside the core construct. For border line items ( I – CVI 0.70 – 0.77 or CVR 0.50 – 0.61), three prespecified criteria were applied:

- Theoretical essentiality – whether the item addressed an otherwise uncaptured facet within its domain;
- Qualitative expert consensus supporting the item’s importance despite borderline ratings;^10^ and
- Structural necessity – whether removal would reduce domain coverage below three items per factor.^12^ Items meeting at least two criteria were retained with revision, others were removed. This was applied to PPHS06 and PPHS 12 (reverse keyed items retained for acquiescence bias mitigation and unique facet coverage).

## RESULTS

Across the expert panel, the scale-level content validity was strong (S-CVI/Ave = 0.88), confirming the content validity of the final 36 items of PPHS. This indicates that on average 88% of experts rated each item as quite or highly relevant, exceeding the recommended threshold of 0.80 and reflecting near consensus that the items appropriately represent the construct domain of public health professionalism.^10^

S-CVI/Ave values at the domain level were also high and consistent (0.83-0.90) (Table-II). Ethical Stewardship and Integrity (Domain 1) and Self-Awareness and Adaptive Professionalism (Domain 6) had the highest scores (S-CVI/Ave = 0.90 each). Despite being the lowest, the Accountability and Professional Duty domain (Domain 2) remained above the commonly accepted threshold level (S-CVI/Ave = 0.83).

**Table-II:** Scale-Level Content Validity Index (S-CVI/Ave) by Domain.

Domain	Items (k)	S-CVI/Ave
D1: Ethical Stewardship & Integrity	6	0.90
D2: Accountability & Professional Duty	6	0.83
D3: Competence & Reflective Growth	7	0.87
D4: Interprofessional Collaboration & Respect	6	0.88
D5: Advocacy & Community Orientation	5	0.86
D6: Self-Awareness & Adaptive Professionalism	6	0.90
Overall Scale	36	0.88

***Note:*** N = 10 experts; recommended threshold S-CVI/Ave ≥0.80.

### Item-Level Content Validity:

Item-level evaluation used I-CVI (relevance ) and CVR (necessity). Applying a more conservative thresholds (I-CVI ≥0.78; CVR ≥0.62), majority of retained items showed high relevance and essentiality agreement.

A few items remained even though their CVR was below the critical value. These items addressed specific domain facets where their removal would have adversely affected domain representation, and the reverse-keyed items was judged to mitigate acquiescence bias (e.g. PPHS06 and PPHS12).

Nine items were removed from the original 45-item pool (Table-III), leading to 80% retention. The main reasons cited for removal were redundancy (n = 5), low relevance (n = 2), lack of specificity (n = 1), and content outside the core professionalism construct (n = 1).

**Table-III:** Items Removed During Content Validation (n=9).

Item	Original Item Text	Reason for Removal
Q7	I accurately represent data and findings, even when they are inconvenient	Redundant with Q4 (ethical reporting)
Q21	I mentor junior colleagues to support their professional growth	Low I-CVI; not core to professionalism construct
Q22	I use feedback to make concrete improvements in my work	Redundant with Q16/Q19
Q26	I provide constructive feedback that supports others’ learning	Overlap with mentoring/collaboration items
Q29	I acknowledge the contributions of other team members	Consolidated into Q21 (share credit)
Q34	I contribute to initiatives that strengthen the public-health system	Too vague; low content validity ratings
Q35	I use my professional role to amplify community voices	Overlap with advocacy items
Q43	I balance my professional commitments to maintain personal well-being	Outside core professionalism construct
Q45	I learn from setbacks and use them to improve my future performance	Redundant with Q39 (constructive criticism)

### Experts’ narrative comments were grouped into four themes:

Wording and clarity (23 items): Suggestions focused on making behaviors more concrete, reducing jargon, and using language commonly used by Pakistani public health professionals. Several items were rewritten in the active voice to improve readability.

### Redundancy and overlap (nine items):

Items perceived as duplicative were consolidated into broader statements or removed to reduce respondent burden without weakening domain coverage.

### Cultural and contextual fit (five items):

Experts recommended wording that better reflected local realities, including resource constraints, hierarchical work environments, and typical modes of community engagement in Pakistan.

### Double-barrelled wording (four items):

Items assessing more than one behavior were simplified to a single construct or separated into clearer statements to improve interpretability.

## DISCUSSION

In the expert panel study, PPHS showed strong content validity for measuring the professionalism among public health professionals in Pakistan (S-CVI/Ave = 0.88). All domain level indices were at acceptable levels, indicating that the item pool sufficiently captures the six dimensions of professionalism.^16^,^17^ Domain level indices (0.83 – 0.90) confirm that no single domain showed inadequate content representation, a prerequisite for subsequent factor analytic work.^18^

Of particular interest, the domains of Ethical Stewardship, Integrity and Self-Awareness, and Adaptive Professionalism, received notably high levels of agreement.^19^,^20^ This may indicate prominence of reflective, ethical, decision-making when professionals are required to justify population-based decisions under various constraints.^21^

From a methodological point of view, the combination of indices and written feedback strengthened the refinement process.^22^,^23^ Indices helped identify weak items, while comments clarified whether problems concerned phrasing, conceptual fit or overlap, etc. This approach is encouraged in the measurement literature and increases transparency in item selection.^24^,^25^ While the CVR criterion is a helpful guide, its application can be overly stringent and may eliminate theoretically relevant items in a given context.

In this study, a small number of borderline items were kept using pre - specified criteria (explained in methodology) to retain domain breadth and preserve reverse-keyed items designed to mitigate response set bias.^12^ By concentrating on public health rather than clinical practice, this work directly addresses the gaps identified in previous reviews of professionalism instruments.^5^,^6^

### Limitations:

A few limitations of the study are as under: First, the 10 expert panel, all based in Pakistan while exceeding recommended minimum standards (5 – 6 experts) for content validation, limits the precision of CVI estimates and may not fully capture different perspectives within public health practice.^10^,^14^ Second, the analysis did not include inter-rater reliability indices (e.g., Fleiss’ kappa) or intraclass correlation coefficients were not calculated and should be included in future research. Third, the panel was drawn exclusively from Pakistani institutions which may introduce cultural bias The exclusively Pakistani panel may introduce cultural bias; cross cultural validity could be achieved by including international expert. Fourth, qualitative feedback was analyzed descriptively rather than through formal thematic analysis. Finally, content validity represents only one of Massick’s five sources of validity evidence whereas the scales psychometric properties require additional evidences such as response process, internal structure and criterion related validity in subsequent phases.

Future phases include response process through cognitive interviews (n = 10 – 15) to verify item interpretation and time to complete the scale, pilot testing (n = 30 – 50) for item analysis, exploratory factor analysis (n = 360; 10 individuals per item)^26^, confirmatory factor analysis on another ssample (n = 300 – 400) to test the six factor structure, reliability assessment (Crohnbach’s alpha, composite reliability, test – retetst) and measurement invariance testing across professional subgroups.^27^

## CONCLUSION

With this structured expert review, the PPHS achieved strong content validity. The 36-item version serves as a contextually grounded foundation for future psychometric evaluation, including pilot testing, response process verification, and internal structure analyses.

### Authors Contribution

**SFM:** Conceived, designed and did statistical analysis & editing of manuscript, is responsible for integrity of research.

**KQ, MAK**: Did data collection and manuscript writing.

**RA:** Did review and final approval of manuscript.
